# Platelet derived exosomes disrupt endothelial cell monolayer integrity and enhance vascular inflammation in dengue patients

**DOI:** 10.3389/fimmu.2023.1285162

**Published:** 2024-01-03

**Authors:** Sayali Vedpathak, Archana Sharma, Sonali Palkar, Varsha R. Bhatt, Vishwanath Chandrashekhar Patil, Arjun L. Kakrani, AkhileshChandra Mishra, Deepak Bhosle, Vidya A. Arankalle, Shubham Shrivastava

**Affiliations:** ^1^ Department of Communicable Diseases, Interactive Research School for Health Affairs (IRSHA), Bharati Vidyapeeth (Deemed to be University), Pune, India; ^2^ Department of Community Medicine, Bharati Vidyapeeth (Deemed to be University) Medical College and Hospital, Pune, India; ^3^ Department of Clinical Immunology and Rheumatology, Bharati Vidyapeeth (Deemed to be University) Medical College and Hospital, Pune, India; ^4^ Department of Critical Care Medicine, Bharati Vidyapeeth (Deemed to be University) Medical College and Hospital, Pune, India; ^5^ Department of Medicine, Dr. D. Y. Patil Medical College Hospital & Research Centre, Dr. D .Y. Patil Vidyapeeth, Pune, India; ^6^ Department of Medicine, Bharati Vidyapeeth (Deemed to be University) Medical College and Hospital, Pune, India

**Keywords:** dengue virus, platelets, exosomes, platelet-derived exosomes, vascular inflammation, sVCAM-1

## Abstract

**Background:**

Thrombocytopenia is the most notable phenomenon in dengue. Activation status of platelets and interaction of platelets with endothelium contribute towards dengue disease pathogenesis. Platelets are the major cell types known to release extracellular vesicles, especially exosomes in circulation. However, the role of platelet derived exosomes (PLT-EXOs) in endothelial dysfunction during dengue infection remains unknown.

**Methods:**

In this study, we recruited 28 healthy subjects and 69 dengue patients categorized as WS- (n=31), WS+ (n=29) and SD (n=9). Platelets were isolated from platelet rich plasma of dengue patients and their activation was assessed by flow cytometry. PLT-EXOs were isolated by ultracentrifugation method. Western blot analyses were performed to characterize the exosomes. Exosome uptake experiment was carried out to see the internalization of exosomes inside endothelial cells (HUVECs). To observe the effect of exosomes on endothelial cells, exosomes were added on HUVECs and expression of adherens and tight junctional proteins were examined by immunofluorescence assay and western blot. Expression levels of vascular injury markers were measured in the culture supernatants of Exosome-HUVEC coculture and sera of dengue patients by MSD-multiplex assay.

**Results:**

As compared to healthy subjects, CD41/CD61 expression was significantly reduced (p<0.0001) and CD62p expression was significantly increased (p<0.0001) on platelets in dengue patients. PLT-EXOs isolated from the dengue patients showed higher expression of CD63 and CD9 proteins than the healthy subjects. With *in-vitro* immunofluorescence assays, we illustrated the internalization of PLT-EXOs by the HUVECs and observed disruption of endothelial cell monolayer integrity in the presence of PLT-EXOs from WS+ and SD patients. Furthermore, the significant reduction in the expressions of ZO-2, VE-Cadherin and CD31 in endothelial cells following exposure to PLT-EXOs from the dengue patients provide direct evidence of PLT-EXOs mediated vascular permeability. PLT-EXOs stimulated the release of inflammatory markers CRP, SAA, sVCAM-1 and sICAM-1 in the supernatants of HUVEC cells. Importantly, significantly higher levels of CRP, sVCAM-1 and sICAM-1 in the sera of severe than mild dengue patients (p<0.0001) suggest their role in disease severity.

**Conclusions:**

In summary, our data suggest that PLT-EXOs promote vascular leakage via release of proinflammatory mediators and compromise vascular barrier integrity in dengue patients.

## Introduction

Dengue is one of the most common mosquito-borne viral infections in the tropical and subtropical countries across the world. In the last 20 years, the disease has steadily expanded with an estimated infection of 3.97 billion people in 129 countries (1). Dengue causes a wide range of clinical manifestations from asymptomatic infections to mild fever without warning signs (WS-), fever with warning signs (WS+) and severe dengue (SD). Severe dengue is characterized by one or more symptoms like plasma leakage, fluid accumulation, respiratory distress, severe bleeding or organ impairment (2, 3). The risk to develop SD has been attributed to multiple factors like dengue virus (DENV) serotype, secondary infection by heterologous serotype, comorbidity, viral load and the host immune response (4–6). Yet, the understanding of the pathogenesis of severe dengue remains unclear.

Severe dengue is associated with transient increase in vascular permeability and plasma leakage due to endothelial cell dysfunction (5, 7). Endothelial cells line the blood vessels, capillaries and form a selective physical barrier for the exchange of molecules through the cells. Dengue NS1 protein has been shown contributing directly to the vascular leakage by degrading sialic acid and heparan sulfate proteoglycans of glycocalyx lining the endothelium (8, 9). Subsequently, raised levels of hyaluronic acid, heparin sulfate and syndecan-1 were shown to be associated with disease severity and mortality in dengue patients (10). Certain cytokines such as TNF-α and IL-1β have also shown to directly cause vascular leakage (11, 12).

Sufficient number of platelets are required to maintain the vascular hemostasis. In dengue patients, thrombocytopenia is frequently observed and is linked with disease severity (13, 14). Thrombocytopenia mainly occurs due to either decreased production of platelets in the bone marrow and/or increased destruction and clearance of platelets from peripheral blood (15). Moreover, the activation status of platelets during DENV infection determines the platelet clearance by phagocytic cells. *In-vitro* study further revealed increased adhesion of DENV activated platelets to endothelium contributing to thrombocytopenia in dengue patients (14, 16). In addition, activated platelets release extracellular vesicles *via* CLEC2 receptor present on the platelets (17). These extracellular vesicles contain chemokines, CCL5 and cytokines, IL-1β to exert their action. Platelets and platelet derived microparticles release IL-1β by activating inflammasome pathway and induce endothelial permeability (13). Even, the overexpression of platelet activating factor was shown to reduce the expression of tight junctional protein, ZO-1 and enhance vascular permeability in dengue (18). This suggests that platelets play a critical role in increased vascular permeability during dengue infection. Other pro-permeability factors like TNF-α, serotonin and VEGF were also shown to induce endothelial permeability and higher levels of these permeability factors were positively correlated with disease severity in dengue patients (18). In this study, we aimed to examine the role of platelet derived exosomes in vascular dysfunction and their association with disease severity in dengue patients.

## Materials and methods

### Ethics statement

Blood samples were taken from dengue patients admitted to the Bharati Vidyapeeth Medical College & Hospital and Dr. D. Y. Patil Medical College Hospital & Research Centre. The study was approved by the Institutional Ethics Committee. Written informed consent was received from all the subjects prior to blood collection.

### Clinical samples

Blood samples were collected from dengue patients hospitalized in Bharati Vidyapeeth Medical College & Hospital and Dr. D. Y. Patil Medical College Hospital & Research Centre. A total of 97 samples were collected including 28 samples from healthy individuals and 69 dengue patients within 10 days after onset of the disease. All sera samples were tested for NS1 antigen (J Mitra, Cat no IR031096), anti-DENV-IgM (Panbio, Cat no 01PE20) and anti-DENV Capture IgG (Panbio, Cat no 01PE10) by ELISA.

### Platelet count

Platelet count was taken at the time of sample collection from dengue patients admitted to the hospital. This count was obtained from the CBC reports of the blood samples tested from dengue patients.

### Platelet preparation

All blood samples were processed within 2 hours after collection. 5ml of whole blood was drawn in Acid Citrate Dextrose (ACD) vacutainer tube. Platelet rich plasma (PRP) was collected by centrifugation at 400 x g for 10 minutes with 0 brake. To avoid the activation of platelets, 100 ng of PGE2 (Sigma, Cat no 5640) was added to PRP. To isolate platelets, PRP was centrifuged at 2000 x g for 10 minutes. Supernatant was collected and further used for exosome isolation and platelet pellet was resuspended in Dulbecco’s Phosphate Buffered Saline (DPBS) (Hi-media, Cat no TL1006) containing 100 ng of PGE2. Platelet purity was checked with flow cytometry.

### Flow cytometry assay

To examine the expression of cell surface markers on platelets, platelets were labeled with immunofluorescence antibodies BV510 CD45 (Cat no 368526, clone 2D1), PerCP-Cy5.5 CD41/CD61 (Cat no 359814, clone A2A9/6) and BV785 CD62P (Cat no 304942, clone AK4) and incubated at 4°C for 30 minutes. Platelets were washed with PBS and then acquired using Cytoflex LX (Beckman Coulter, USA).

### Exosome purification

To isolate exosomes secreted from platelets, collected supernatant was centrifuged at 26500 x g for 30 minutes at 4°C using Optima-X-100 (Beckman Coulter, USA) with SW55ti rotor. Pellet was used as microvesicles fraction for immunoblotting and supernatant was further centrifuged at 110,000 x g for 70 minutes to pellet down the exosomes. After removal of supernatant, pellet containing exosomes was resuspended in DPBS. After two ultracentrifugation steps, each at 110,000 x g for 60 minutes, exosome pellet was washed with DPBS in each step. The exosome pellet obtained after final ultracentrifugation step was stored at -80°C for further use.

### Particle size distribution

Exosomes were diluted 1:100 in distilled water and then exosome size was captured using Dynamic light scattering (DLS) instrument NANOPHOX Sympatec (GmbH).

### Immunoblotting

Cell lysates were prepared with RIPA buffer (1M NaCl, 1% NP40, 0.5% sodium deoxycholate, 0.1% sodium dodecyl sulphate, 50mM Tris of pH 7.4 and protease inhibitors). Platelet lysates from pellet, microvesicles and exosomes fractions from PRP of healthy subjects and dengue patients, exosomal proteins (10µg) and HUVEC lysates (20µg) were separated by SDS PAGE and proteins were transferred to 0.2 μm nitrocellulose membrane. Membranes were then incubated overnight with primary antibodies - CD63 (Cat no. sc5275 - 1:1000, Santa Cruz), CD9 (Cat no. 13147S - 1:1000, Cell Signaling Technology), CD41 (Cat no. 13807S - 1:1000, Cell Signaling Technology), GAPDH (Cat no. G9545 - 1:5000, Sigma), VE-Cadherin (Cat no. 2158S - 1:1000, Cell Signaling Technology), CD31 (Cat no. 3582S - 1:1000, Cell Signaling Technology), ZO-2 (Cat no. 2847S - 1:1000, Cell Signaling Technology). After three subsequent washes of nitrocellulose membrane with TBST, membranes were incubated with peroxidase-conjugated anti-mouse secondary antibody (Cat no. 115-035-003 - 1:7500, Immuno Jackson) or anti-rabbit secondary antibody (Cat no. 111-035-003 - 1:7500, Immuno Jackson) and blots were developed using enhanced chemiluminescence reagent (Cat no. 1705060, Bio-Rad).

### Cell culture

HUVECs (Human Umbilical Cord Vein Endothelial Cells) were purchased from Thermofisher Scientific (Cat no. C0035C). Cells were maintained in M200 media (Cat no. M200500, Thermofisher Scientific) supplemented with Large Vessel Endothelial supplement (LVES) (Cat no. A14608-01, Thermofisher Scientific) and incubated at 37°C with 5% CO_2_ atmosphere.

### Labeling and internalization of exosomes

PLT-EXOs were labeled with PKH67-cell membrane labeling kit (Cat no. MIDI67, Sigma) according to the manufacturer’s protocol. Briefly, 150μl exosomes were mixed with 850μl of diluent C containing 6μl of PKH67 dye. After 5 minutes of incubation at room temperature, 2 ml of 10% BSA was added to the PKH67 labeled exosomes to quench the labeling process. This 3ml of labeled exosome and BSA mixture was added on the top of 1ml of 15% sucrose solution and labelled exosomes were pellet down by ultracentrifugation at 1,90,000 x g for 2 hrs. Supernatant was discarded and exosome pellet was transferred to Amicon 10 KDa MWCO filter column (Cat no. UFC901024D, Sigma). Total volume adjusted to 10 ml with DPBS and centrifuged at 3000 x g for 40 minutes and then concentrate was recovered. 2x10^4^ cells were seeded in 8-well collagen coated slides (Cat no. 80809, iBiDi) and following day, PKH67 labeled exosomes were incubated with HUVECs for 2 hours at 37°C with 5% CO_2_ atmosphere.

### *In-vitro* incubation of exosomes with endothelium cells

50μg of PLT-EXOs isolated from blood samples with different clinical presentations were added on the HUVECs (0.25x10^6^ cells/well in 6 well plate) previously coated with 2% gelatin and incubation was done for 48 hrs.

### Immunofluorescence assay

HUVECs were fixed with 3.7% formaldehyde for 20 minutes, washed once with PBS and then permeabilized with 0.2% triton X-100 for 10 minutes followed by two washes with PBS. Cells were blocked with 3% BSA for 60 minutes and then two PBST washes were given to the cells. Primary antibodies Claudin -1 (Cat no. sc166338, Santa Cruz) and VE-Cadherin (CST) were used in 1:200 dilution with 0.1% BSA and cells were incubated with these antibodies overnight at 4°C. After completion of 2 washes with PBST, fluorescently labeled secondary antibodies anti-mouse Alexa-fluor 488 (Cat no. A11029) and anti-rabbit Alexa-fluor 546 (Cat no. A11035) (1:200 dilution with 0.1% BSA) (Invitrogen) were added on the cells for 60 minutes. After two washes, the nucleus was stained with DAPI. Images were acquired using ZEISS AXIO fluorescence microscope under 20X objective.

### Endothelial permeability assay

For permeability assay, upper layer of transwell chamber (0.4 µm, Cat no. 353495 Falcon) was seeded with the 0.5x105 HUVECs/100µL and in the lower chamber, 600µL M200 media was added for 24 hrs. 2% Evans blue dye stock was prepared and filtered. 0.6 mg/ml Evans-blue containing 4% BSA working solution was prepared for the assay. Next day, PLT-EXOs were incubated with the HUVECs in 2% M200 containing exosome depleted serum while the lower chamber was replaced with fresh 600 µL 2% M200 media for 24 hrs. After 24 hrs, previous media from the lower chamber was aspirated and upper chamber was filled with 100µL of Evans blue working stock solution and incubated for 10 minutes. Then, the 200µL media from the lower chamber was used for the measurement of the O.D. at 620nm using microplate reader. (Synergy HTX, Biotek, USA).

### MSD-multiplex assay

Vascular injury panel 2 (human) kit (Cat no. K15198D, MSD) that measures concentrations of four inflammation markers CRP, SAA, sVCAM-1 and sICAM-1 was used for multispot ELISA using Meso Scale Discovery (MSD, Germany) instrument. Serum samples (1:1000 diluted) and undiluted culture supernatants of HUVECs collected from PLT-EXO-HUVECs coculture experiment were used for the study. The assay was performed using MSD 96-well 4 spot assay plate in duplicates according to the manufacturer’s instructions.

### Statistical analysis

All data was expressed as the mean ± standard error. Statistical analysis was carried out using proportion test, unpaired t-test and non-parametric Mann-Whitney test to compare the two groups. Proportion test was used to compare the percentage differences between gender, and clinical parameters of WS-, WS+ and SD patients with healthy subjects. For the comparison of normalized expression of PLT-EXOs protein markers and endothelial surface markers, unpaired t-test was performed. Mann-Whitney test was used for comparison of clinical parameters like platelet count, hemorrhagic manifestations, percent expression of platelet surface markers, and secretion of inflammatory markers between two groups.

For correlation analyses, non-parametric Spearman correlation-regression plot with the 95% interval was performed. To predict the potential of sVCAM-1 as biomarker, ROC curve was plotted by calculating area under the curve.

GraphPad Prism software (Version 10.0, GraphPad Software, Inc., San Diego, CA, USA) was used to perform the data analysis. p-value less than 0.05 was considered statistically significant.

## Results

### Clinical features of dengue patients

A total of 69 patients positive for one or more dengue seromarkers were recruited for this study (**Table 1**). Of these, 11.6% were positive for NS1 antigen only, 40.6% were positive for both NS1 and anti-DENV IgM and 47.8% were positive for anti-DENV IgM only. Based on capture IgG ELISA, 23 patients had primary, and 46 patients had secondary dengue infection. Following WHO 2009 guideline, dengue patients were categorized based on the symptoms at the time of admission to the hospital (**Table 2**). Out of 69 dengue patients, 31 were classified as mild patients (WS-) with fever, chills, headache, nausea and arthralgia. 29 dengue patients were categorized as WS+ with additional complications like hemorrhagic manifestations and vascular permeability signs during their hospital stay. Abdominal pain was prominent in WS+ patients (p<0.0001). All the nine severe dengue patients showed serious disease complications including organ involvement. Platelet count of <150,000/μL was detected in 60 (86.96%) patients at the time of admission. Hemorrhagic manifestations were observed in 13 patients (18.8%) of which 11 patients (84.6%) had severe thrombocytopenia with platelet count less than 50,000/μL (**Table 2**). Significant reduction in platelet count with less than 50,000/μL was noted in 75.9% of WS+ (p<0.0001) and 88.9% of SD (p<0.0001) when compared with WS- dengue patients.

**Table 1:** Clinical features of the study groups.

Study groups*	Healthy subjects (n=28)	Dengue patients (n=69)
Median Age, (range in years)	23 (20–39)	30 (4–76)
Gender		
Female	18 (64.3)	25 (36.2)
Male	10 (35.7)	44 (63.8)
Dengue confirmatory tests (%)		
NS1 positive	0	8 (11.6)
NS1 & IgM dual positive	0	28 (40.6)
IgM positive	0	33 (47.8)
Primary Dengue	N.A.	23 (33.3)
Secondary Dengue	N.A.	46 (66.7)

*Data are expressed as number (%) or else specified. N.A. denotes not applicable.

**Table 2:** Clinical symptoms of dengue patients.

Disease Category*	Dengue (%)	WS- (%)	WS+ (%)	SD (%)	P value^$^	P value^@^	P value^#^
Probable dengue signs^1^	69 (100)	31 (44.93)	29 (42.03)	9 (13.04)	N.A.	N.A.	N.A.
Warning signs symptoms							
Abdominal pain or tenderness	17 (24.6)	0 (0)	14 (48.3)	3 (33.3)	<0.0001	0.008	0.68
Median Platelet count per µL (Interquartile range)	56000 (28000-102500)	95000 (75000-164000)	31000 (21000-49000)	28000 (15500-34000)	N.A.	N.A.	N.A.
Platelet count < 50,000/µL	30 (43.5)	0 (0)	22 (75.9)	8 (88.9)	<0.0001	<0.0001	0.7
Hemorrhagic manifestations^2^	13 (18.8)	0 (0)	10 (34.5)	3 (33.3)	0.001	0.008	1
Vascular permeability indications^3^	3 (4.4)	0 (0)	1 (3.5)	2 (22.2)	0.44	0.06	0.4
Signs of organ damage^4^	9 (13.04)	0 (0)	0 (0)	9 (100)	N.A.	<0.0001	<0.0001

*Data are expressed as number (%) or else specified. WS- represents dengue patients without warning signs, WS+ represents dengue patients with warning signs, SD denotes severe dengue patients, N.A. denotes not applicable, ^$^indicates statistical significance of comparison of WS- with WS+ patients, ^@^denotes statistical significance of comparison of WS- with SD patients and ^#^represents statistical significance of comparison of WS+ and SD patients. ^1^combinations of one or more symptoms such as fever, chills, arthralgia, headache, nausea and/or vomiting; ^2^petechiae, bleeding, epitaxis, hematuria, hypoalbumenia, malena; ^3^plasma leakage indicated by ascites, pleural effusion; ^4^splenomegaly, liver injury, seizure, higher AST or ALT, transaminitis, respiratory distress, gall bladder wall thickening, hepatic failure etc.

### Disease severity in dengue patients relates to the alteration of platelet surface markers

**Figure 1:**
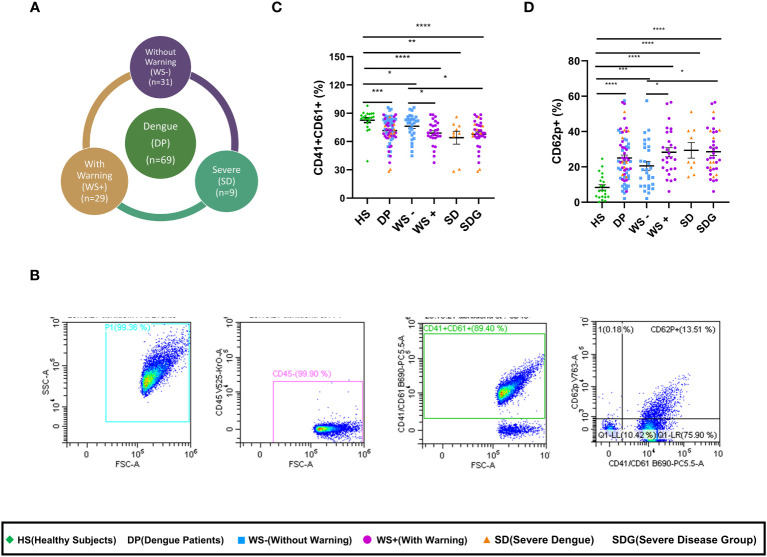
Alteration of platelet surface markers in different clinical presentations of dengue patients. **(A)** Pie chart showing number of dengue patients of different categories as per WHO 2009 classification. **(B)** Gating strategy used to assess the platelet activation status in dengue patients and healthy subjects. Expression levels of **(C)** CD41/CD61 and **(D)** CD62p on platelets of healthy subjects (n=21) and dengue patients (n=65) of different clinical presentations, WS- (n=29), WS+ (n=27) and SD (n=9). HS denotes healthy subjects, DP denotes dengue patients, WS- denotes dengue patients without warning signs, WS+ denotes dengue patients with warning signs, SD represents severe dengue patients and SDG represents severe disease group. Data is represented as mean ± standard error. All the samples were analyzed by non-parametric Mann-Whitney test in which * represents p < 0.05, ** represents p < 0.01, *** represents p < 0.001 and **** represents p < 0.0001.

To assess platelet activity in dengue infection, we quantitated expression levels of platelet surface markers in dengue patients presenting with different clinical manifestations. **Figure 1A** shows the proportion of dengue patients with different clinical presentations. As the number of severe dengue patients was less (n=9), the patients were categorized as mild dengue i.e., WS- and severe dengue group (SDG) that consisted of dengue patients with warning signs (WS+) and severe disease (SD). **Figure 1B** shows the gating strategy of the platelet population. After selecting the FSC/SSC total cell population, CD45 negative population was chosen. CD45 negative population was devoid of leukocytes and > 99% of the cell population consisted of platelets. CD41/CD61 dual positive population was then separated from the CD45 negative cell population. Later, the platelet activation marker, p-selectin (CD62p) was plotted against CD41/CD61 dual positive population. As compared to the healthy subjects, the expression of CD41/CD61 on platelet surface was significantly reduced in dengue patients (p=0.0001), WS- (p=0.023), WS+ (p<0.0001), SD (p=0.001) and SDG patients (p<0.0001, **Figure 1C**). Importantly, CD41/CD61 expression was significantly reduced in WS+ and SDG patients than WS- group (p=0.029, p=0.014 respectively, **Figure 1C**).

Next, the activation status of platelets in dengue patients was assessed by measuring P-selectin (CD62p) expression levels. As compared to the healthy subjects, CD62p expression on platelet surface was significantly upregulated in overall dengue patients (p<0.0001, **Figure 1D**) as well as in mild, WS- (p=0.0002) and severe disease, WS+, SD and SDG (p<0.0001 for all). A significant rise in CD62p levels in the WS+ and SDG than WS- patients (p=0.025, p=0.01, **Figure 1D**) suggests that the disease severity was linked to platelet activation.

In our study groups, a significantly higher number of males were infected with the dengue virus than females (p=0.002). We therefore, examined the association of gender with the alteration of platelet surface markers. The gender-wise comparison revealed similar expression levels of CD41/61 (p=0.49) and of CD62p (p=0.17) in the males and females. This finding indicates that platelet activation is associated with disease severity and independent of gender.

**Figure 2:**
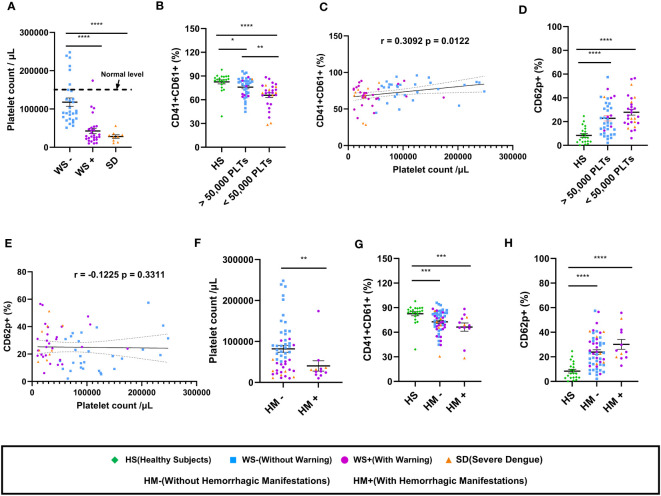
Platelet count and its correlation with platelet surface markers in dengue patients. **(A)** Platelet counts in dengue patients with different clinical presentations, WS- (n=29), WS+ (n=27) and SD (n=9). **(B)** Comparison of levels of CD41/CD61 in healthy subjects (n=21) with dengue patient having platelet count >50,000/μL (n=37) and dengue patient having platelet count <50,000/μL (n=28) respectively. **(C)** Correlation analyses of thrombocytopenia with CD41/CD61 surface expression on platelets. **(D)** Comparison of levels of CD62p in healthy subjects (n=21) with dengue patient having platelet count >50,000/μL (n=37) and dengue patient having platelet count <50,000/μL (n=28) respectively. **(E)** Correlation analyses of thrombocytopenia with CD62p surface expression on platelets. **(F)** Comparison of platelet counts in dengue patients without (HM-, n=53) and with hemorrhagic manifestations (HM+, n=12). Comparison of levels of **(G)** CD41/CD61 and **(H)** CD62p in healthy subjects (n=21) with dengue patients without (HM-, n=53) and with hemorrhagic manifestations (HM+, n=12). HS denotes healthy subjects, WS- denotes dengue patients without warning signs, WS+ denotes dengue patients with warning signs, SD represents severe dengue patients, HM- represents dengue patients without hemorrhagic manifestations and HM+ represents dengue patients with hemorrhagic manifestations. Data is represented as mean ± standard error. Data were analyzed by non-parametric Mann-Whitney test between the two groups. Correlation analyses were performed using two-tailed Spearman’s correlation coefficient test. * represents p < 0.05, ** represents p < 0.01, *** represents p < 0.001 and **** represents p < 0.0001.

Next, we examined the association of platelet count with the expression of platelet surface markers in dengue patients (**Figure 2**). As expected, total platelet count reduced with disease severity showing a significant reduction in the SD and WS+ patients than the WS- category (p<0.0001, **Figure 2A**). Reduced surface expression of CD41/CD61 was notable in the dengue patients with platelet count lesser than 50,000/μL than in healthy subjects (p<0.0001, **Figure 2B**). CD41/CD61 expression on platelets was drastically reduced in the dengue patients with platelet count <50,000/μL than in the patients with platelet count >50,000/μL (p=0.006, **Figure 2B**). We noted a weak but positive correlation of CD41/CD61 expression on platelet surface with platelet count (r=0.31, p=0.01, **Figure 2C**). In the case of CD62p expression on platelet surface, dengue patients with platelet count lesser than 50,000/μL had higher expression of CD62p than the healthy subjects (p<0.0001, **Figure 2D**). CD62p expression was higher in dengue patients irrespective of the platelet counts. We did not observe any significant correlation between CD62p expressing platelets and thrombocytopenia (**Figure 2E**).

We further examined the expression of platelet surface markers in dengue patients with hemorrhagic manifestations. The platelet count in dengue patients having hemorrhagic manifestations (HM+) was lower than those without hemorrhagic manifestations (HM-) (p=0.007, **Figure 2F**). Comparable levels of surface expression of CD41/CD61 (p=0.19, **Figure 2G**) and CD62p (p=0.15, **Figure 2H**) were recorded in HM- and HM+ dengue patients.

In summary, our results suggest that reduced expression of CD41/CD61 and enhanced expression of platelet activation markers are associated with disease severity. Male predominance among dengue patients had no effect on the alteration of platelet surface markers. Irrespective of hemorrhagic manifestations, reduced expression of CD41/CD61 surface markers was associated with thrombocytopenia in dengue patients.

### Platelet derived exosomes isolated from dengue patients express CD63 and CD9 on their surface

**Figure 3:**
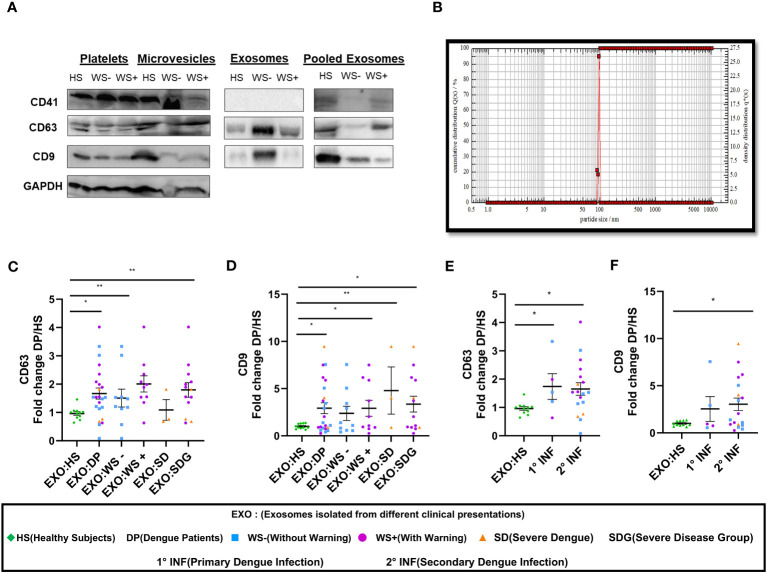
Platelet derived exosomes isolated from dengue patients express CD63 and CD9 proteins. **(A)** Expression of CD41, CD63 and CD9 proteins in pooled fractions (n=5) of platelets-pellet, microvesicles and exosomes isolated from healthy subjects and each category of dengue patients as shown by western blot. **(B)** Size distribution of platelet specific exosomes though DLS. Fold change expressions of **(C)** CD63 and **(D)** CD9 in dengue patients (n=23), WS- (n=10), WS+ (n=10), SD (n=3), SDG (n=13) compared to healthy subjects (n=11). Comparisons of fold change expression of **(E)** CD63 and **(F)** CD9 in exosomes derived from platelets of primary (1° INF, n=5) and secondary (2° INF, n=18) dengue patients with healthy subjects (n=11). HS denotes healthy subjects, DP denotes dengue patients, WS- denotes dengue patients without warning signs, WS+ denotes dengue patients with warning signs, SD represents severe dengue and SDG represents severe disease group. EXO:HS denotes platelet derived exosomes from healthy subjects, EXO:DP denotes platelet derived exosomes from dengue patients, EXO:WS- denotes platelet derived exosomes from dengue patients without warning signs, EXO:WS+ denotes platelet derived exosomes from dengue patients with warning signs, EXO:SD denotes platelet derived exosomes from severe dengue and EXO:SDG denotes platelet derived exosomes from severe disease group. All the samples were analyzed by parametric unpaired t test in which * represents p < 0.05 and ** represents p < 0.01.

Next, we studied the role of platelet derived exosomes in dengue disease severity. First, we detected CD41, the platelet specific marker and exosomal markers CD63 and CD9 in the platelet fractions - pellet, microvesicles and exosomes isolated from platelet rich plasma (PRP) of healthy subjects and dengue patients suggesting that platelets and platelet derived exosomes (PLT-EXOs) express exosome specific proteins (**Figure 3A**). CD41 expression was only seen in pooled exosomes from 5 subjects of each category but not in the individual PLT-EXOs from all categories reflecting the low concentration of exosomes in protein fraction. Then, from a subset of samples, exosomes were isolated from PRP through ultracentrifugation. These purified exosomes were first characterized by Dynamic Light scattering (DLS) where 99% of exosomes were shown to be approximately 100 nm in size (**Figure 3B**). On immunoblotting, differential expression of CD63 and CD9 proteins was observed in the PLT-EXOs derived from dengue patients with different clinical presentations (**Figure 3A**). On quantitation, fold change expression of CD63 and CD9 in PLT-EXOs was significantly higher in all the dengue patients (p=0.02, p=0.03), WS+ (p=0.001, p=0.029) and SDG (p=0.008, p=0.02) respectively than the healthy subjects (**Figures 3C, D**). Fold change expressions of CD63 (p=0.24) and CD9 (p=0.45) were comparable in exosomes isolated from WS- and SDG groups suggesting that expression levels of exosome markers were not correlated with disease severity. Similar expression levels of CD63 and CD9 were seen when exosomes from primary and secondary dengue patients were compared (**Figures 3E, F**).

We next quantitated the PLT-EXOs in dengue patients by measuring the total PLT-EXOs protein amount (per mL of PRP) in samples used for western blotting. We observed comparable levels of exosome protein content in HS (n=8, 330 ± 92) and in dengue patients of different categories, WS- (n=10, 359 ± 119, p=0.89), WS+ (n=10, 177 ± 29, p=0.32) and SD (n=3, 1022 ± 386, p=0.08). The non-significant value for the SD group may be because of the small number. This preliminary data with a smaller number of samples strongly suggests that exosome protein content may be higher in the SD patients but need to be confirmed in the subsequent studies.

In summary, our results suggest that PLT-EXOs isolated from dengue patients express CD63 and CD9 at significantly higher levels than healthy subjects and disease severity is associated with the increased secretion of PLT-EXOs in dengue patients.

### Loss of cell adhesion molecules in the endothelium owing to the internalization of platelet derived exosomes

**Figure 4:**
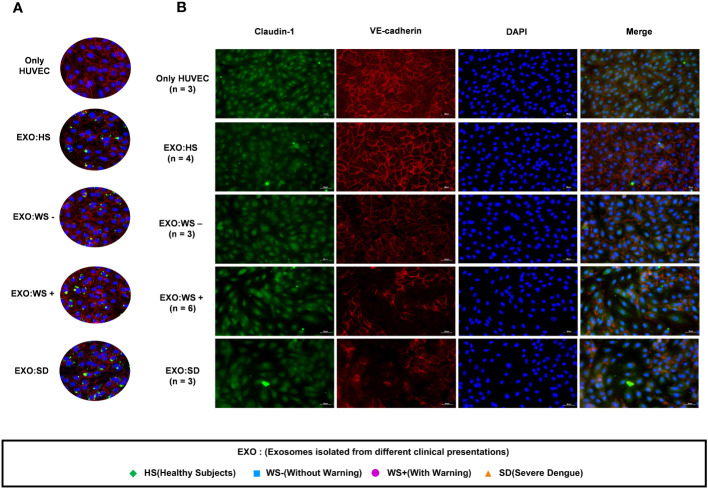
Effect of platelet derived exosomes on endothelial cells. **(A)** Internalization of exosomes isolated from platelets of healthy subjects and dengue patients of each categories (WS-, WS+ and SD). **(B)** Immunofluorescence images of HUVECs showing expression of cell adhesion molecules Claudin-1 (green) and VE-Cadherin (Red) with nucleus stained with DAPI (blue) on exposure of platelet derived exosomes isolated from dengue patients for 48 hr onto HUVEC cells. Only HUVEC denotes untreated HUVEC cells, EXO:HS denotes platelet derived exosomes of healthy subjects, EXO:WS- denotes platelet derived exosomes from dengue patients without warning signs, EXO:WS+ denotes platelet derived exosomes from dengue patients with warning signs and EXO:SD represents platelet derived exosomes from severe dengue patients. n denotes the number of patients from which platelet derived exosomes of different categories were tested on HUVEC cells. Representative images were shown here.

As exosomes are known to play a critical role in intercellular communication, we examined the effect of platelet derived exosomes (PLT-EXOs) in endothelial cells. PKH67 labeled exosomes were taken up by the HUVECs within 2 hours of incubation irrespective of the origin of the exosomes (**Figure 4A**). Next, we examined the expression profile of cell adhesion proteins, Claudin-1 and VE-Cadherin, on exposure of exosomes to HUVECs by immunofluorescence assay. HUVEC monolayer remained intact when PLT-EXOs isolated from healthy subjects were added onto endothelial cells. Importantly, on exposure to PLT-EXOs isolated from WS+ and SD patients, a significant loss of adhesion molecules was evident (**Figure 4B**).

**Figure 5:**
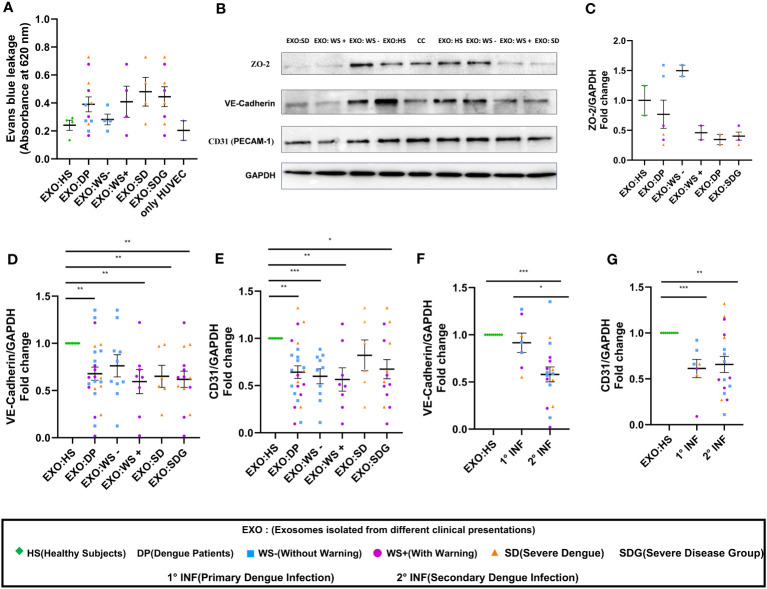
Vascular dysfunction associated with platelet derived exosomes in dengue patients. **(A)** Transwell assay of endothelial permeability using BSA-Evans blue dye. Graphical representation of absorbance readings at 620 nm by plotting optical density (OD) values of microtiter plate of transwell treated with PLT-EXOs isolated from healthy subjects and dengue patients (n=4 from each category). **(B)** Expression of ZO-2, VE-cadherin, CD31 on exposure of platelet derived exosomes from healthy subjects and dengue patients after 48 hr in HUVEC cells as shown by Immunoblotting. **(C)** Fold change expressions of ZO-2 in HUVEC cells after treatment of exosomes isolated from different clinical presentations (n=2 from each category). Fold change expressions of **(D)** VE-Cadherin and **(E)** CD31 in HUVEC cells after treatment of exosomes isolated from HS (n=9), DP (n=24), WS- (n=10), WS+ (n=8), and SD (n=6). Comparisons of fold change expression of **(F)** VE-Cadherin and **(G)** CD31 in HUVEC cells after addition of exosomes derived from primary (1° INF, n=7) and secondary (2° INF, n=17) dengue patients with healthy subjects (n=11). CC denotes untreated HUVEC cells. EXO:HS denotes platelet derived exosomes from healthy subjects, EXO:DP denotes platelet derived exosomes from dengue patients, EXO:WS- denotes platelet derived exosomes from dengue patients without warning signs, EXO:WS+ denotes platelet derived exosomes from dengue patients with warning signs, EXO:SD denotes platelet derived exosomes from severe dengue and EXO:SDG denotes platelet derived exosomes from severe disease group. Data is represented as mean ± standard error. Fold change expression analyses were carried out using parametric unpaired t test in which * denotes p < 0.05, **denotes p < 0.01 and *** denotes p < 0.001.

Next, we performed BSA-Evans blue leakage assay to examine the permeability of HUVEC on the addition of PLT-EXOs from dengue patients. Due to limited sample availability, PLT-EXOs from 4 individual subjects of each category were used for the experiment. We noted leakage of Evans blue dye in the lower chamber with increased OD value in transwell inserts treated with PLT-EXOs derived from WS+ (2/4) and SD (3/4) patients than healthy subjects (**Figure 5A**). Due to the low sample size, no significant difference in permeability was seen between healthy and dengue patient groups. However, our results indicate that PLT-EXOs from the severe disease category induced the permeability of endothelial cells.

Next, we compared the expressions of ZO-2, VE-Cadherin, and CD31 in HUVECs following exposure of PLT-EXOs isolated from healthy subjects or dengue patients by immunoblotting. The expression of all three cell adhesion molecules was reduced after the addition of PLT-EXOs isolated from WS+ or SD patients (**Figure 5B**). Due to the limited sample availability, we could only perform immunoblotting of ZO-2 in six dengue patients, two from each category. The expression of ZO-2 in HUVEC cells was dependent on the source of the exosomes and was reduced in the severe disease group (**Figure 5C**). As compared to healthy controls, fold change expression of VE-Cadherin and CD31 were significantly reduced in dengue patients (p=0.009, p=0.003) as well as, WS+ (p=0.004, p=0.002) or SDG (p=0.002, p=0.02) groups (**Figures 5D, E**). Fold change expressions of VE-Cadherin and CD31 were comparable among WS- and SDG (p=0.32, p=0.59) (**Figures 5D, E**). We found significantly reduced expression of VE-Cadherin in the secondary than primary dengue patients (p=0.03, **Figure 5F**); CD31 expressions remained unchanged (p=0.78, **Figure 5G**).

In summary, our results pointed direct role of PLT-EXOs released from severe dengue patients in inducing endothelial permeability. PLT-EXOs reduced the expression of adhesion and junction proteins and promoted vascular leakage by damaging the integrity of endothelial cells.

### Platelet derived exosomes from dengue patients contribute to vascular inflammation

**Figure 6:**
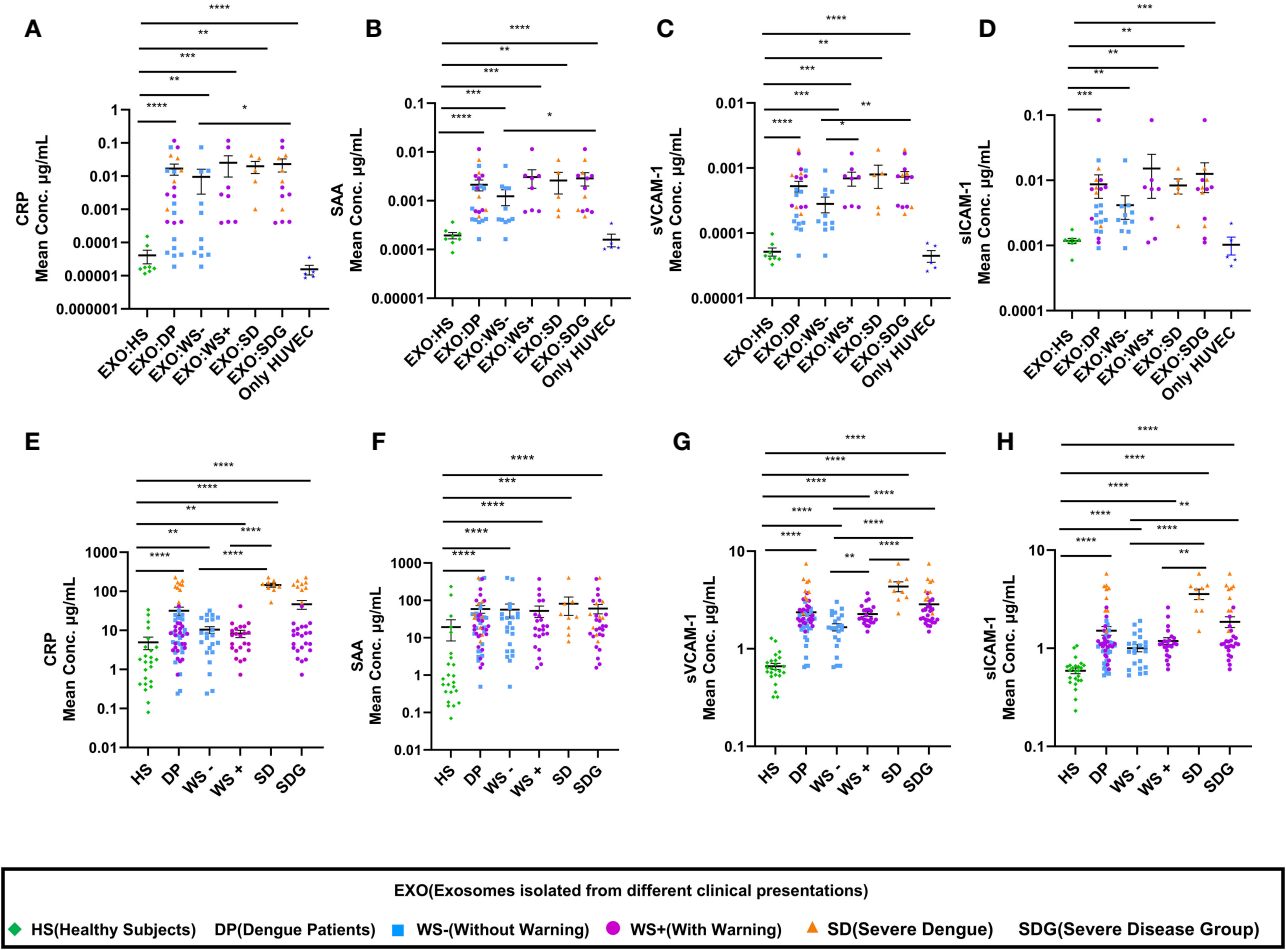
Enhancement of vascular injury markers in dengue patients through platelet derived exosomes. **(A-D)** Comparisons of CRP, SAA, sVCAM-1, sICAM-1 levels in supernatants of exosome-HUVEC coculture of EXO:HS (n=8), EXO:DP (n=24), EXO:WS- (n=11), EXO:WS+ (n=8), EXO:SD (n=5) with untreated HUVEC cells (n=5). **(E-H)** Comparisons of CRP, SAA, sVCAM-1, sICAM-1 levels in serum samples of healthy subjects (n=24) and dengue patients (n=54) with different clinical presentations, WS- (n=22), WS+ (n=23), SD (n=9). EXO:HS denotes platelet derived exosomes from healthy subjects, EXO:DP denotes platelet derived exosomes from dengue patients, EXO:WS- denotes platelet derived exosomes from dengue patients without warning signs, EXO:WS+ denotes platelet derived exosomes from dengue patients with warning signs, EXO:SD+ denotes platelet derived exosomes from severe dengue and EXO:SDG denotes platelet derived exosomes from severe disease group. Only HUVEC denotes untreated HUVEC cells. HS denotes healthy subjects, DP denotes dengue patients, WS- denotes dengue patients without warning signs, WS+ denotes dengue patients with warning signs, SD represents severe dengue patients and SDG represents patients in severe disease group. Data is represented as mean ± standard error. Mean concentrations were analyzed by non-parametric Mann-Whitney test in which * represents p < 0.05, ** represents p < 0.01, *** represents p < 0.001 and **** represents p < 0.0001.

Inflammation is one of the major causes of endothelial dysfunction. Hence, we examined the direct effect of PLT-EXOs in vascular inflammation. **Figures 6A–D** displays the soluble levels of the vascular injury markers (CRP, SAA, sVCAM-1 and s-ICAM-1) in the supernatants obtained following the incubation of HUVECs with PLT-EXOs derived from various study groups. As compared to healthy controls, the levels of all the four vascular injury markers were significantly higher in the dengue patients (CRP, SAA and sVCAM-1: p<0.0001 and sICAM-1: p=0.0002). Notably, a significantly higher expression levels were observed in the SDG than WS- patient groups for CRP and SAA (p=0.035); and sVCAM1 (p=0.006) respectively (**Figures 6A–C**). These results suggest that the induction of inflammation in vascular endothelium by platelet derived exosomes is a characteristic of dengue infection and dependent on the severity of disease.

Further, we extended our *in-vitro* observations to clinical dengue (**Table 3**). For this, circulating levels of the vascular injury markers in the sera of dengue patients with different clinical presentations were compared. Notably, the serum levels of all the four markers were significantly higher in the dengue patients than the healthy individuals (p<0.0001, **Figures 6E–H**) suggestive of their role in the pathogenesis of the disease. Of prime importance, disease severity specific differences were identified. CRP, sVCAM-1 and sICAM-1 could differentiate between mild (WS-) and severe disease groups (WS+ and SD) (**Figures 6E, G, H**). sVCAM-1 emerged to be the single marker differentiating between WS-, WS+ and SD patients and needs to be explored further as a biomarker for dengue disease severity. Though SAA rise was characteristic of dengue infection, the levels were independent of disease severity (**Figure 6F**).

**Table 3:** Levels of vascular inflammatory markers in sera of dengue patients.

Vascular Injury Markers	HS (n=24)	DP (n=54)	WS- (n=21)	WS+ (n=24)	SD (n=9)	P value^&^	P value^$^	P value^@^	P value^#^
CRP Mean conc. (µg/mL)	4.96 ± 1.74	31.79 ± 7.47	10.39 ± 2.05	8.28 ± 1.76	144.2 ± 16.57	<0.0001	0.71	<0.0001	<0.0001
SAA Mean conc. (µg/mL)	19.22 ± 11.02	58.97 ± 13.69	56.69 ± 23.01	52.46 ± 17.92	81.20 ± 41.41	<0.0001	0.97	0.16	0.2
sVCAM-1 Mean conc. (µg/mL)	0.66 ± 0.05	2.37 ± 0.17	1.66 ± 0.14	2.27 ± 0.11	4.35 ± 0.5	<0.0001	0.002	<0.0001	<0.0001
sICAM-1 Mean conc. (µg/mL)	0.59 ± 0.04	1.51 ± 0.15	1.0 ± 0.08	1.18 ± 0.09	3.57 ± 0.4	<0.0001	0.12	<0.0001	<0.0001

Data is represented in Mean ± SEM. P values were calculated by non-parametric Mann Whitney test. ^&^ indicates statistical significance between HS and DP ^$^indicates statistical significance of comparison of WS- with WS+ patients, ^@^denotes statistical significance of comparison of WS- with SD patients and ^#^represents statistical significance of comparison of WS+ and SD patients.

**Figure 7:**
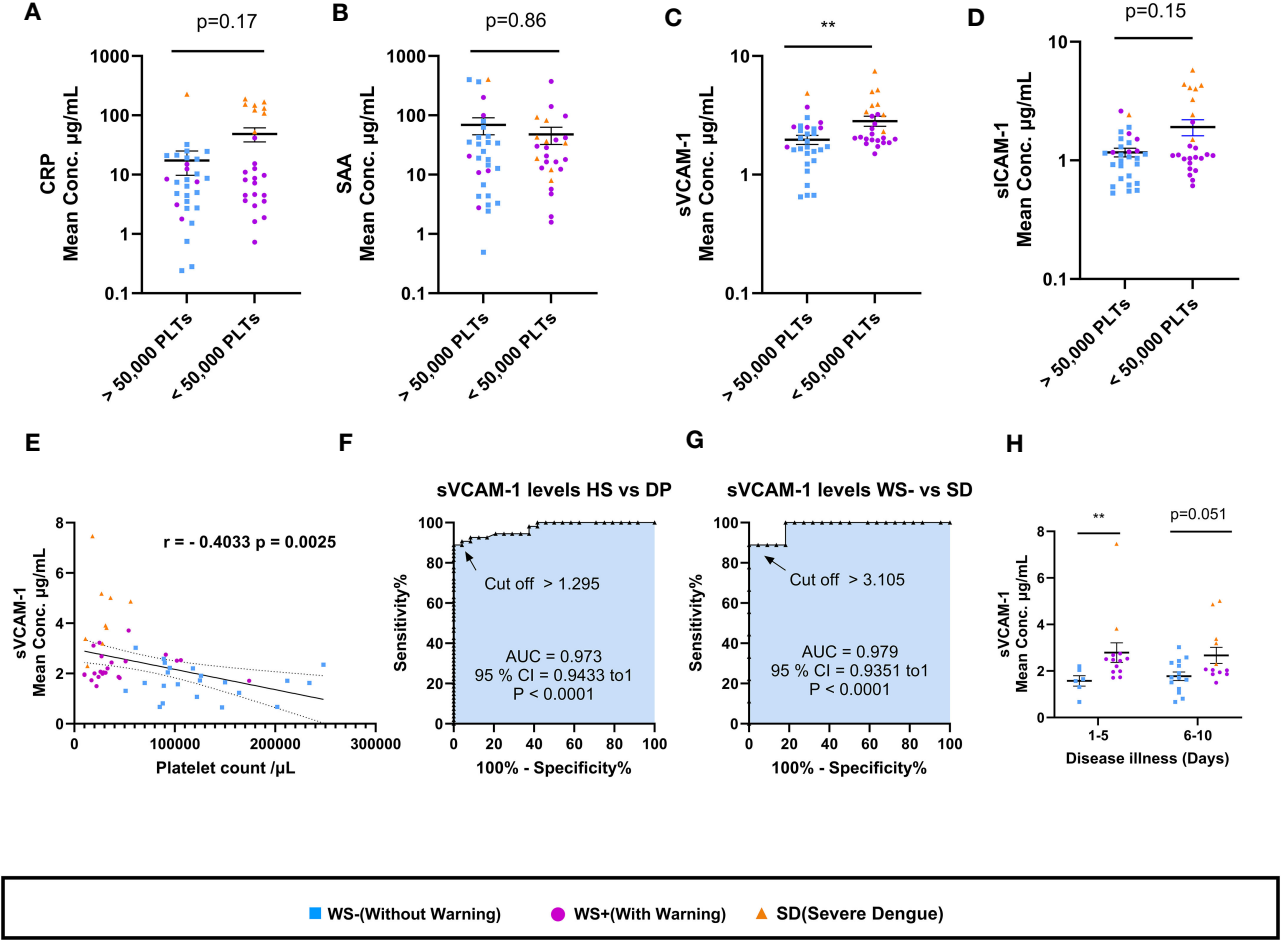
Association of sVCAM-1 levels with thrombocytopenia and disease severity. **(A-D)** Comparisons of levels of CRP, SAA, sVCAM-1 and sICAM-1 in dengue patients having platelet count >50,000/µL (n=29) with dengue patients having platelet count <50,000/µL (n=25). **(E)** Correlation analysis of sVCAM-1 levels in sera of dengue patients with platelet counts. ROC curve presentations differentiating sVCAM-1 levels of **(F)** healthy subjects with dengue patients and **(G)** WS- with SD patients. **(H)** sVCAM-1 levels in sera of WS- and severe disease group of WS+ and SD patients at different days post onset of illness. At POD 1-5, n= 6 for WS- patients and n=13 for SDG respectively. At POD 6-10, n=14 for WS- patients, and n=12 for SDG respectively. HS denotes healthy subjects, DP denotes dengue patients, WS- denotes dengue patients without warning signs, WS+ denotes dengue patients with warning signs and SD represents severe dengue patients. Data is represented as mean ± standard error. Mean concentrations were analyzed by non-parametric Mann-Whitney test in which ** represents p < 0.01. Correlation analysis was performed using two-tailed Spearman’s correlation coefficient test.

In view of the importance of platelet count in dengue disease severity and management, the four vascular inflammatory markers were compared in relation to platelets (**Figure 7**). Strikingly, only sVCAM-1 levels were significantly higher in dengue patients having platelet count <50,000/µL (p=0.007, **Figure 7C**). In addition, moderate negative correlation of sVCAM-1 with thrombocytopenia is noteworthy (r = -0.40, p = 0.0025; **Figure 7E**). The levels of CRP, SAA, sVCAM-1 and sICAM-1 were similar in dengue patients with or without hemorrhagic manifestations (**Supplementary Figure 1**). With the limited number of patients enrolled in this study, we explored sVCAM-1 as a predictive biomarker of severe dengue infection (**Figures 7F–G**). At a concentration of 1.295μg/mL, sVCAM1 could distinguish between the healthy subjects and dengue patients with 89% sensitivity, 100% specificity (AUC=0.973, **Figure 7F**). At a concentration of 3.105 μg/mL, sVCAM1 could distinguish between the WS- and severe dengue patients with 89% sensitivity, 100% specificity (AUC=0.979, **Figure 7G**). On comparison of sVCAM-1 levels at different time points post onset of illness (POD), sVCAM-1 levels were significantly higher in severe disease group at POD 1-5 (p=0.005, **Figure 7H**) and maintained at higher levels on POD 6-10 than mild dengue patients. This suggests that elevated levels of sVCAM-1 could be the marker of disease severity in early stage of infection. Taken together, sVCAM-1 levels were linked with thrombocytopenia and disease severity in dengue patients.

## Discussion

**Figure 8:**
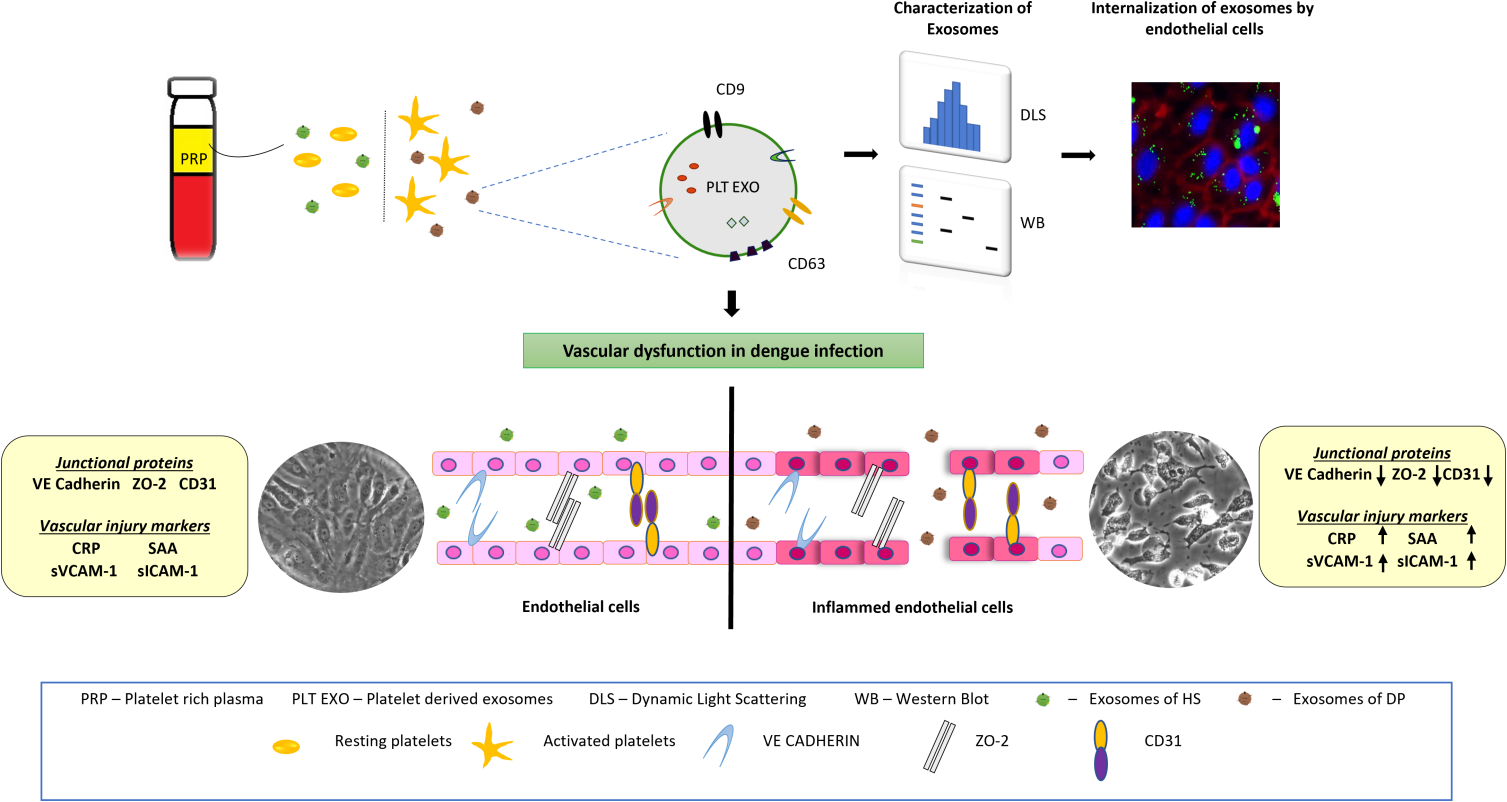
Schematic representation showing the effect of platelet derived exosomes on endothelial cells in dengue patients. Dengue virus activates platelets which in turn release exosomes on to the bloodstream. Endothelial cells internalize exosomes which carry CD63 and CD9 proteins. After internalization, platelet secreted exosomes damage endothelial cells integrity and induce inflammation. Inflamed endothelial cells subsequently increases the disease severity in dengue patients.

For the first time, our study provides direct evidence for the role of platelet secreted exosomes derived from dengue patients in affecting vascular endothelium leading to impairment of endothelial cell barrier integrity, increased permeability and inflammation. Exposure of PLT-EXOs isolated from dengue patients reduced the expression of Claudin-1, CD31 cell adhesion molecules, VE-Cadherin and ZO-2 intercellular junctional proteins of the endothelium and stimulated the excessive release of inflammatory markers from endothelial cells. Moreover, elevated levels of vascular injury markers in the sera of dengue patients suggest PLT-EXOs mediated vascular inflammation in dengue patients (**Figure 8**).

Severe dengue was characterized by thrombocytopenia, hemorrhage and vascular leakage (6, 19). As per the WHO guidelines, a platelet count below the level of 150,000/μL of blood is considered thrombocytopenia (20). We and several other groups have reported thrombocytopenia as the clinical indicator of disease severity in dengue patients (**Table 2**). In our study, 88.9% of severe dengue and 75.9% of WS+ patients had platelet count below 50,000/μL of blood. We also noted severe thrombocytopenia below 50,000/μL in dengue patients with hemorrhagic manifestations (10/12, 83%) (p=0.003, **Figure 2F**). High frequency of bleeding was reported in the patients with severe thrombocytopenia with platelet count of less than 20,000/μL of blood (21). On the contrary, we had only 3/17 (18%) patients with bleeding complications and platelet counts of less than 20,000/μL of blood. The lower platelet counts below 50,000/μL of blood were associated with the reduced expression of CD41/CD61 (**Figure 2B**). In the patients with Glanzmann thrombasthenia (22, 23) and acute myeloid leukemia (24), low levels of the CD41-CD61 complex resulted in reduced platelet aggregation and thereby, increased bleeding disorders. However, in our patient series, CD41/CD61 expression was similar in dengue patients irrespective of hemorrhagic manifestations (**Figure 2G**). The reason for this remains unclear.

In earlier studies, platelet activation was reported either during the febrile phase (13) or during the critical phase of dengue infection (25). While confirming platelet activation observed by others (13, 14, 25), our study noted enhanced platelet activation in relation to disease severity (**Figure 1D**). During a longitudinal study, platelet activation was shown to be inversely correlated with platelet counts (14). In the absence of follow-up samples, we could not generate such data.

Dengue virus (DENV) can enter platelets probably through DC-SIGN and heparan sulfate proteoglycan co-receptors but fails to replicate and reproduce infectious virus particles (26–28). However, exposure to DENV directly activates platelets. Upon activation, platelets release two types of extracellular vesicles i.e., (EVs), exosomes and microvesicles (MVs), in circulation (29–31). *In-vitro* data clearly showed that only activated platelets can release EVs and the number of EVs increased in an MOI-dependent manner after incubation with dengue virus. DENV-induced release of EVs was higher than thrombin or aggretin activated platelets (17). Dengue virus activates CLEC2 surface receptor on platelets to release EVs comprising of both exosomes and microvesicles (17). We characterized these PLT-EXOs isolated from dengue patients presenting with different clinical manifestations. As expected, these PLT-EXOs were of ~100nm in size, and expressed CD63 and CD9 proteins on their surface. Both the proteins were also present on the activated platelets from dengue patients. An earlier *in-vitro* study revealed similar levels of CD63 and CD9 expression in EVs after incubation with dengue virus (17). Of note, CD63 and CD9 expression levels were significantly higher on the PLT-EXOs isolated from dengue patients than the healthy subjects (**Figures 3C, D**). This difference was not evident when CD63 and CD9 expression on PLT-EXOs were compared among WS-, WS+ and SD groups probably due to limited sample size. In dengue patients, erythrocytes and platelets were identified as the major cell populations releasing EVs, especially MVs in circulation (32). Shedding of EVs was dependent upon the active viral replication and degree of apoptotic cell death (14, 32). Absolute count and the percentage of platelet derived MVs were significantly lower in DHF patients during the febrile phase of infection that was correlated with thrombocytopenia and bleeding disorders (32). In this study, we did not measure the absolute counts of platelet derived exosomes from dengue patients and therefore, correlation between the absolute counts of PLT-EXOs with disease severity could not be established.

Platelets play a pivotal role in maintaining vascular homeostasis. On activation, platelets not only release EVs but also enhance the interaction with neutrophils and other immune cells like mast cells and macrophages to promote inflammation associated vascular permeability (17, 33–35). However, whether activated platelets and PLT-EXOs directly contribute to vascular permeability is still unknown. Our findings of significant reduction in the expression of endothelial cell adhesion molecules, (Claudin-1, CD31), intercellular junctional proteins (VE-Cadherin, ZO-2) and Evans blue dye leakage in transwell inserts by PLT-EXOs isolated from WS+ and SD patients provide direct evidence for the role of PLT-EXOs in vascular permeability (**Figure 5**). Importantly, irrespective of disease severity, PLT-EXOs isolated from dengue patients reduced the expression of intercellular junctional proteins by more than 1.5-fold (**Figures 5C–E**). In contrast, the integrity of endothelial cells remained intact on exposure to PLT-EXOs derived from healthy subjects. PLT-EXOs from human PRP are known to carry several analytes like growth factors and chemokines even under basal conditions (36), however, their effect on endothelial cells was not studied. Even in dengue patients, PLT-EXOs are not yet fully characterized. In one of the earlier reports, MVs isolated from DENV-2 exposed platelets were shown to cause endothelial permeability (13). These platelet derived MVs carried IL-1β and treatment with IL-1β receptor agonist were able to abrogate permeability induced by platelet derived MVs.

In this study, PLT-EXOs were derived directly from PRP of dengue patients and therefore, the presence of dengue NS1 protein inside the PLT-EXOs could not be ruled out. Indeed, we could detect NS1 protein inside the PLT-EXOs isolated from the primary dengue patients. However, out of 24 PLT-EXOs isolated from dengue patients, 50% were derived from NS1 negatives and still exhibited enhanced vascular permeability. It is likely that NS1 did aid in enhancing vascular permeability but was undetectable at the time of sample collection. It is plausible that PLT-EXOs may carry matrix metalloproteinases (MMPs) and other pro-permeability factors like TNF-α, serotonin, PAF, and microRNA to regulate the integrity of endothelial cells. However, platelet derived exosomes of dengue patients are yet to be characterized. It seems that both viral proteins, as well as host factors may have contributed to endothelial dysfunction. Our clinical findings confirm earlier observations of overexpression of NS1 protein directly contributing to vascular leakage (8, 9). Furthermore, PLT-EXOs enhanced the secretion of vascular injury markers, CRP, SAA, sVCAM-1 and sICAM-1 after exposure to endothelial cells (**Figure 6**). This suggests that PLT-EXOs promoted vascular leakage *via* the release of proinflammatory mediators leading to compromised vascular barrier integrity in dengue patients.

Endothelial cell activation is characterized by the increased expression of ICAM-1 and VCAM-1 on the cell surface or may circulate as soluble molecules as a result of proteolytic cleavage from the cell membrane (37, 38). Several groups have reported endothelial cell activation with high levels of circulating ICAM-1 and VCAM-1 in dengue patients (39–41). Subsequently, levels of sICAM-1 together with sVCAM-1 were found to be elevated in sera of dengue patients as compared to patients with other febrile illnesses (42). Particularly, sVCAM-1 and sICAM-1 levels were elevated in DHF patients (39–41). We provide a comparative and comprehensive analysis of sVCAM-1 and sICAM-1 levels in sera of dengue patients presenting with different clinical manifestations. Significantly higher levels of sVCAM-1 and sICAM-1 were observed in the sera of severe dengue patients than in patients with WS+, WS- and healthy individuals (**Figure 6**). In addition, elevated levels of sVCAM-1 at 3 µg/mL could distinguish mild from severe dengue patients. Koraka et al. (40), also reported significant difference in the levels of sVCAM-1 levels among mild and DSS patients but not with DHF patients. With respect to disease duration, significantly elevated levels of sVCAM-1 levels in severe than mild dengue patients were noted till 10-12 days post onset of illness in a study from China (43) and till 5 days post onset of illness in this study (**Figure 7H**). Although, significant difference in the levels of sVCAM-1 was observed between mild and severe dengue patients, the prognostic value of sVCAM-1 as biomarker needs further evaluation in a prospective longitudinal study with large sample size. Furthermore, similar to the findings of Murgue et al. (39), the levels of sVCAM-1 and sICAM-1 were comparable in sera of primary and secondary dengue patients (**Supplementary Figure 2**).

CRP has been identified as a biomarker for differentiating dengue from other febrile illnesses and predicting severe disease (44, 45). In our study, 8/9 (88.9%) severe dengue patients had more than 100 mg/L levels of CRP (**Table 3**). Here, two independent reports are noteworthy. High CRP levels were observed in severe dengue patients of >100mg/L in all six DSS patients including 2 fatalities (44). The other study documented a 20-fold rise in 9 patients with severe disease (46). We did not find any difference among patients with or without warning signs suggesting CRP to be a marker for severe disease as reported earlier. High CRP levels indicate tissue damage due to higher inflammation levels and are often associated with poor outcome. Another prominent acute phase response protein in response to inflammation is hepatocyte derived SAA. High levels of SAA were observed in sera of dengue patients irrespective of disease severity. Elevated levels of SAA were reported in other inflammatory diseases like rheumatoid arthritis, atherosclerosis, Crohn’s disease and type 2 diabetes patients (47).

In summary, we report for the first time that in dengue patients, platelet derived exosomes (PLT-EXOs) mediate vascular inflammation. In dengue patients, the exposure of platelets to the virus or NS1 antigen in circulation leads to platelet activation and alteration of platelet adhesion molecules. The activated platelets release exosomes characterized by the presence of CD63 and CD9 on their surface. PLT-EXOs disrupts the endothelial cell barrier integrity and enhance the secretion of inflammatory markers. Reduced platelet count further aggravates the vascular inflammation and enhance disease severity. Taken together, platelet derived exosomes may act as a supplementary host factor influencing clinical outcome in dengue patients by aggravating vascular permeability.

## Data availability statement

The original contributions presented in the study are included in the article/**Supplementary Material**. Further inquiries can be directed to the corresponding author.

## Ethics statement

The studies involving humans were approved by Institutional Ethics Committee, Bharati Vidyapeeth Medical College & Hospital, Pune and Dr. D. Y. Patil Medical College Hospital & Research Centre, Pune. The studies were conducted in accordance with the local legislation and institutional requirements. Written informed consent for participation in this study was provided by the participants’ legal guardians/next of kin.

## Author contributions

SV: Formal analysis, Methodology, Software, Writing – original draft. AS: Data curation, Methodology, Writing – original draft. SP: Data curation, Resources, Writing – original draft. VB: Data curation, Resources, Writing – original draft. VP: Resources, Writing – original draft. AK: Resources, Writing – original draft. AM: Resources, Writing – review & editing. DB: Resources, Writing – original draft. VA: Writing – review & editing. SS: Conceptualization, Funding acquisition, Project administration, Supervision, Writing – review & editing.

## Supplementary material

Supplementary Figure 1 Association of vascular injury markers with hemorrhagic manifestations in dengue patients. Comparisons of levels of **(A)** CRP, **(B)** SAA, **(C)** sVCAM-1 and **(D)** sICAM-1 in dengue patients without (HM-, n= 42) or with hemorrhagic manifestations (HM+, n=12). DP denotes dengue patients, HM- represents dengue patients without hemorrhagic manifestations and HM+ represents dengue patients with hemorrhagic manifestations. Data is represented as mean ± standard error. Data were analyzed by non-parametric Mann-Whitney test between the two groups.

Supplementary Figure 2 Expression levels of vascular injury markers in primary and secondary dengue patients. Comparisons of levels of **(A)** CRP, **(B)** SAA, **(C)** sVCAM-1 and **(D)** sICAM-1 in primary (1° INF, n=19) and secondary (2° INF, n=35) dengue patients. DP denotes dengue patients. Data is represented as mean ± standard error. Data were analyzed by non-parametric Mann-Whitney test between the two groups.
