# Supplementary figures and images for: Platelet derived exosomes disrupt endothelial cell monolayer integrity and enhance vascular inflammation in dengue patients
Source: Front Immunol. 2024 Jan 3;14:1285162. doi: 10.3389/fimmu.2023.1285162 (PMC10791899; doi:10.3389/fimmu.2023.1285162)

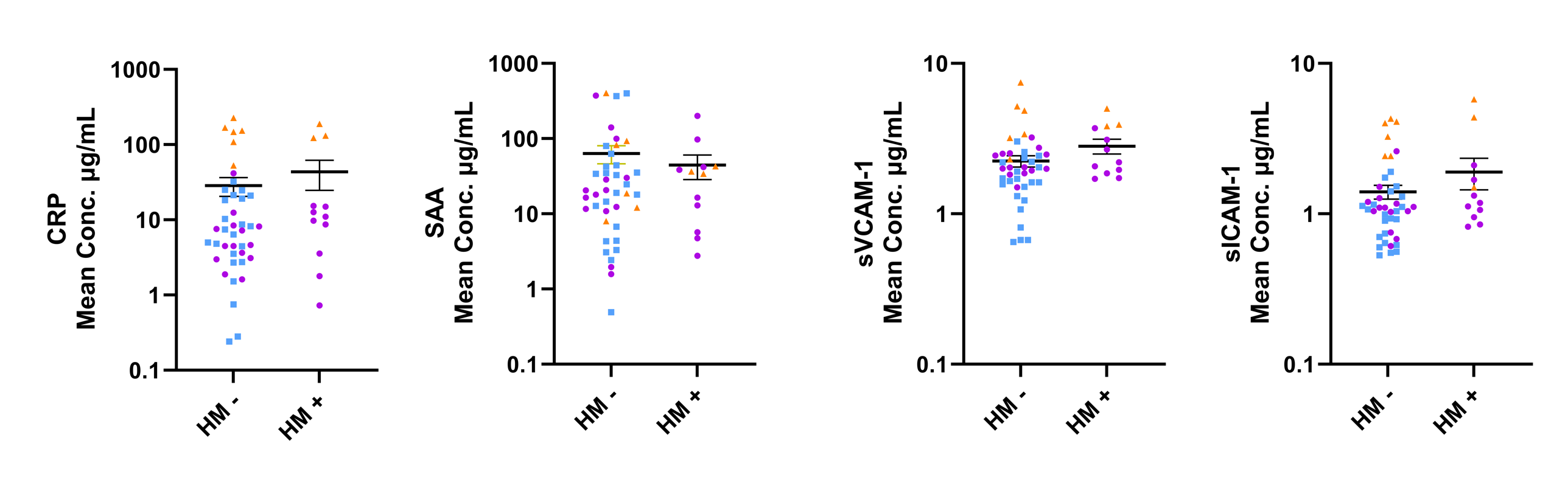

Supplement: Supplementary Figure 1 — Association of vascular injury markers with hemorrhagic manifestations in dengue patients. Comparisons of levels of (A) CRP, (B) SAA, (C) sVCAM-1 and (D) sICAM-1 in dengue patients without (HM-, n= 42) or with hemorrhagic manifestations (HM+, n=12). DP denotes dengue patients, HM- represents dengue patients without hemorrhagic manifestations and HM+ represents dengue patients with hemorrhagic manifestations. Data is represented as mean ± standard error. Data were analyzed by non-parametric Mann-Whitney test between the two groups. [file Image_1.tif]

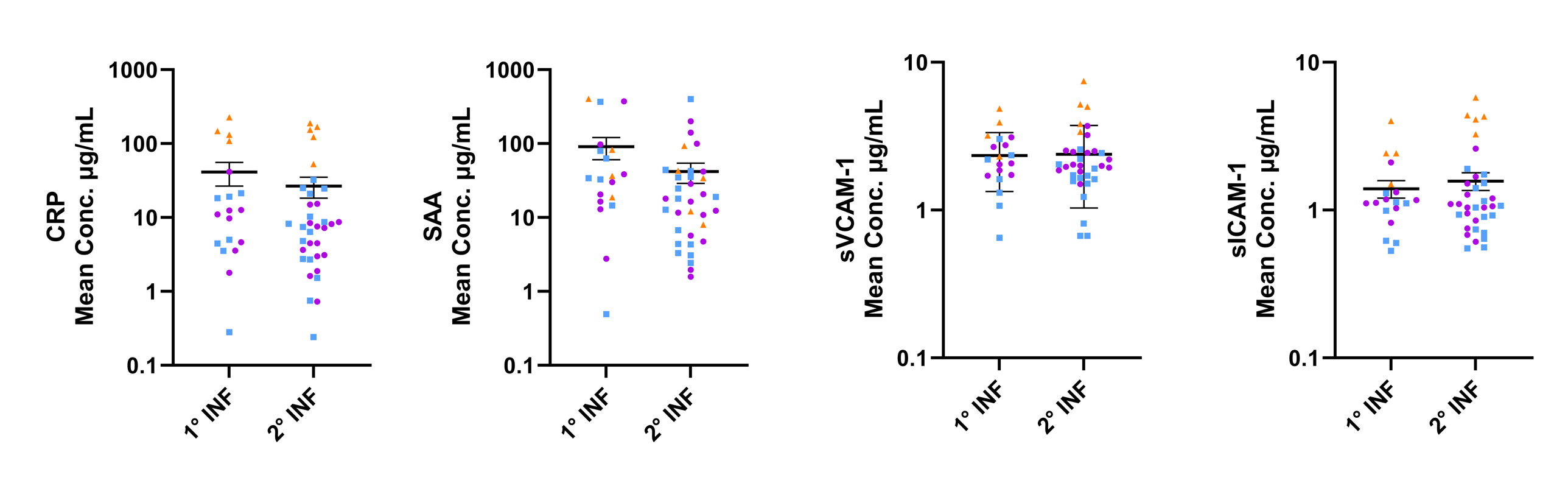

Supplement: Supplementary Figure 2 — Expression levels of vascular injury markers in primary and secondary dengue patients. Comparisons of levels of (A) CRP, (B) SAA, (C) sVCAM-1 and (D) sICAM-1 in primary (1° INF, n=19) and secondary (2° INF, n=35) dengue patients. DP denotes dengue patients. Data is represented as mean ± standard error. Data were analyzed by non-parametric Mann-Whitney test between the two groups. [file Image_2.tif]
